# Malondialdehyde-acetaldehyde (MAA) modified proteins induce pro-inflammatory and pro-fibrotic responses by liver endothelial cells

**DOI:** 10.1186/1476-5926-2-S1-S25

**Published:** 2004-01-14

**Authors:** Geoffrey M Thiele, Michael J Duryee, Monte S Willis, Michael F Sorrell, Thomas L Freeman, Dean J Tuma, Lynell W Klassen

**Affiliations:** 1 University of Nebraska Medical Center, Department of Internal Medicine, 983025 Nebraska Medical Center, Omaha, NE 68198-3025, USA; 2 Omaha VA Medical Center, Research Services 151, 4101 Woolworth Avenue, Omaha, NE, 68105, USA

## Introduction

Recent reports have implicated liver non-parenchymal cells in liver injury as they can secrete; pro-inflammatory cytokines, excessive matrix proteins during fibrosis, and have been shown to initiate local immune responses [1-3]. Of the non-parenchymal cells, sinusoidal liver endothelial cells (SLECs) have been suggested to be a major contributor to the inflammatory processes observed in alcoholic liver disease (ALD) [4]. This is because SLECs are involved in the recruitment of leukocytes into the liver following the activation of an immune response, such as that observed in alcoholic hepatitis [4]. This process involves the release of cytokines TNF-alpha and IL-1beta, which increases the expression of adhesion molecules on the surface of SLECs and the release of chemokines (MIP-2 or MCP-1), which recruit leukocytes into the liver from the circulation [5,6]. These chemokines cause increased binding of leukocytes to the vessel walls and promote the trans-migration of these immune cells across the SLECs by the process of diapedesis [7,8].

Recent studies have shown that proteins can be modified by the metabolites of chronic ethanol consumption. Two of these metabolites, malondialdehyde (MDA) and acetaldehyde (AA), have been shown to synergistically bind to proteins (adduct) to form a product termed MAA [9]. This results in the binding and degradation of the adducted protein. However, chronic ethanol consumption decreases their degradation, but not the binding of these MAA modified proteins. Thus, the extended presence and binding of the MAA-modified proteins to their appropriate receptors could result in the induction of some biological response. It is thought that since these adducts are chronically present, a potential inflammatory response may be initiated similar to that observed in chronic viral infections. Additionally, observation of SLECs when exposed to MAA-Alb showed morphological changes in these cells that was suggestive of cell death (personal observation). Preliminary studies suggested that these changes may be due to TNF-alpha secretion by SLECs. Thus, it was the purpose of these experiments to begin assessing whether MAA-modified proteins can activate SLECs.

## Methods

### Rats

Male Wistar rats were purchased from Charles River Laboratories (Wilmington, MA) and maintained on water and laboratory chow ad libitum. Pair-fed animals were placed on nutritionally adequate liquid diets formulated according to the method of Lieber and DeCarli were obtained from Dyets, Inc. (Bethlehem, PA) [10]. All animals were maintained in the animal facility at the Omaha VA Medical Center which is an AALAC accredited institution.

### Chemicals and Proteins

Bovine serum albumin (Alb) was purchased from CalBiochem (La Jolla, CA). Unlabeled acetaldehyde (AA) was obtained from Aldrich Chemical Co. (Milwaukee, WI). Malondialdehyde (MDA) was obtained as the sodium salt (MDA-Na) by treatment of tetramethoxypropane (Aldrich Chemical Co.) with NaOH, according to the method of Kikugawa and Ido [11].

### Preparation and Labeling of Ligands

Formaldehyde treatment of Alb (f-Alb) was done using a modification of the methods of Mego et al. and Horiuchi et al [12,13]. The advanced glycosylated endproduct (AGE) was prepared as previously reported [14]. All MDA, AA, and MAA adducts were prepared as described elsewhere [9]. The protein concentration was determined by the method of Lowry [15]. All samples were monitored for endotoxin using a Limulus Amebocyte Lysate Assay from BioWhittaker (Walkersville, MD).

### Preparation of Sinusoidal Liver Endothelial Cells (SLECs)

Sinusoidal liver endothelial cells (SLECs) were prepared by the perfusion and differential centrifugation methods described previously [16]. Cell number and viability was determined by trypan blue exclusion on a hemocytometer, and viability was routinely greater than 85%. Cell purity was assayed by staining with "specific" markers for endothelial cells which included; mouse anti-RECA-1 (Serotec, Raleigh, NC), mouse anti-ED2 (Serotec, Raleigh, NC), mouse anti-Desmin (Sigma, St. Louis, MO) and Dil-Ac-LDL (Biomedica Technologies Inc., Stoughton, MA).

### Cytokine Assay Kit Analysis

Supernatants were analyzed for TNF-alpha, MCP-1, MIP-2 using cytokine ELISA kits. TNF-alpha and MCP-1 kits were purchased from Pharmingen (San Diego, CA) and MIP-2 was purchased from BioSource International (Carmillio, CA). All ELISA kits were developed, stopped and read at 450 nm on a MR7000 plate reader, and analyzed using the BIOLINX– software. Assays were run at least 5 times using cells from five different sets of rats.

### Immunohistochemical Staining of SLECs

After isolation and ligand stimulation, SLECs were stained for adhesion molecule expression using antibodies to the following adhesion molecules: Factor VIII (Sigma Chemical Co., St. Louis, MO), Vimentin, RT1A (Class I), RT1B (Class II), CD54 (ICAM-1), CD106 (VCAM-1), CD62P (P-Selectin), CD31 (PECAM-1), CD11b (MAC-1) (Pharmingen, San Diego, CA). Samples were analyzed by 3 independent investigators under a Nikon-FX microscope.

### Fluorescent Activated Cell Sorter (FACs) Analysis of Adhesion Molecules

Following incubation with the various ligands, cells were stained for the presence of: CD54 (ICAM-1), RT1A (Class I), RT1B (Class II), CD44H (H-CAM), CD62L (L-selectin), CD106 (VCAM-1), CD31 (PECAM-1), CD62P (P-Selectin), CD80 (B7-1), CD86 (B7-2), CD11b (MAC-1) (Pharmingen, San Diego, CA).

### Detection of Fibronectin and the EIIIA Isoform

Following incubation of the cells with different ligands, the cells were lysed and tested for the presence of total fibronectin utilizing an ELISA. To better identify and quantify the isotype of fibronectin, Western blotting procedures were performed [17] using antibodies to EIIIA and EIIIB.

### Statistical Analysis

Statistics were performed using the Student's t-test comparing the experimental group to a control. Statistical significance was achieved if P values were less than 0.05 (SigmaStat, Jandel, Scientific, 1994).

## Results

### Release of Cytokines/Chemokines Following MAA-Alb Stimulation

In order to determine whether pro-inflammatory cytokines/chemokines were secreted following MAA-Alb stimulation, SLECs were isolated and incubated with; media, Alb, MAA-Alb, and LPS for various periods of time. After 3 hours of exposure to MAA-Alb, 872 ng/ml of TNF-alpha was secreted. In contrast, LPS a known stimulator of TNF-alpha release, induced the secretion of about one-half-as much TNF-alpha (491 ng/ml) after the same period of time. Also, incubation with the negative control (Alb) resulted in the secretion of 112 ng/ml, and this was considered background. Interestingly, background levels of TNF-alpha increased slightly from SLECs isolated from rats chronically consuming ethanol.

The chemokine MCP-1 was secreted following stimulation of SLECs with; MAA-Alb (606 ng/ml), Alb alone (314 ng/ml), or LPS (384 ng/ml). Additionally, 832 ng/ml of MIP-2 chemokine was released by SLECs following 4 hours of MAA-Alb stimulation. The negative control Alb and positive control LPS produced 211 ng/ml and 222 ng/ml, respectively. These data demonstrate that following incubation with MAA-modified proteins, there is an increased secretion of the cytokine TNF-alpha and the chemokines MCP-1 and MIP-2, into the supernatant of cultured SLECs. Additionally, SLECs from isolated ethanol-fed rats could increase their levels of secretion.

### Immunohistochemical Staining for Adhesion Molecules

To begin assessing the up-regulation of adhesion molecules on the surface of SLECs, cells were isolated, plated on fibronectin coated cover slips, and stimulated with MAA-Alb or Alb. Factor VIII (a positive control for endothelial cells) was expressed on both pair-fed and ethanol-fed animal cells. Vimentin expression was increased in SLECs exposed to MAA-Alb in both the pair-fed control and ethanol-fed animals approximately 2 fold over that of native Alb. Also, both MHC Class I and MHC Class II were up-regulated following MAA-Alb stimulation. However, more significantly, the amount of ICAM-1 and VCAM-1 staining observed following the stimulation of SLECs with MAA-Alb was greatly increased. P-selectin and PECAM-1 were only slightly increased on SLECs from pair-fed and ethanol-fed animals following MAA-Alb exposure. Therefore, these data show that adhesion molecule expression is increased following MAA-Alb stimulation, and chronic ethanol consumption increases these levels.

### Adhesion and Co-Stimulatory Molecule Expression

In order to further quantify the number of adhesion molecules expressed on SLECs from chow-fed rats, isolated cells were stained for adhesion molecules and analyzed by FACS, following stimulation with media, Alb, MAA-Alb, and LPS. ICAM-1 expression on SLECs stimulated by MAA-Alb was increased by 10.31 fluorescent units as compared to stimulation by Alb, but this was not significantly different. In contrast, HCAM-1 expression was decreased by 1.48 units. Increased levels of PECAM-1 (7.87 units), VCAM-1 (8.42 units), and L-selectin (11.70 units) was observed following MAA-Alb stimulation. The most significant change was in the expression of P-selectin, which increased by 44.49 units over that observed with Alb alone. These data, showed similar results as observed by using immunohistochemical staining.

No difference in the amount of Class I expression was observed. However, there was a 25.13 unit increase in the amount of Class II expression when comparing the Alb group to the MAA-Alb stimulated SLECs. Additionally, there was an increase in B7-1 (4.88 units) and B7-2 (15.41 units) expression after MAA-Alb stimulation. These data suggest a possible mechanism for the processing and presentation of aldehyde-modified proteins, and the subsequent activation of an immune responses through SLECs.

### Release of Total Fibronectin and the EIIIA Isoform

Recent studies have shown that SLECs may play an active role in the fibrotic/cirrhotic process, as stimulated SLECs can release fibronectin EIIIA that can stimulate stellate cells to release collagen [17]. Following exposure to MAA-Alb, SLECs release 3–4 fold increased levels of total fibronectin as compared to control ligand stimulation. Additionally, Western Blot analysis showed that the increased fibronectin secretion was primarily of the EIIIA isoform. Thus, MAA-Alb has the ability to increase the secretion of EIIIA isoform which could result in the secretion of collagen by stellate cells. Finally, chronic ethanol consumption increased this effect, once again suggesting that failure to remove these product could result in a pro-fibrotic response.

## Discussion

The ability of immune cells to infiltrate the liver in response to a stimulus has profound implications in alcoholic liver disease. This has best been characterized in patients with alcoholic hepatitis, where there is a leukocyte infiltration into the liver parenchyma of immune cells including monocytes, lymphocytes and neutrophils [5,18]. Recently, sinusoidal liver endothelial cells (SLECs) have been shown to be involved in the inflammatory process, as they possess many surface molecules that are necessary for the recruitment and attachment of leukocytes [4,5]. This process is accomplished when cytokines or chemokines are released into the area of damaged tissue, stimulating SLECs to up-regulate adhesion molecules [5]. Additionally, leukocytes are recruited to the liver with their intergins fully expressed. Once these leukocytes reach the SLECs, they are able to bind (adhesion molecules to intergins), the matrix between them loosens, and leukocytes diapedese into the parenchyma.

In yet other studies, SLECs have been shown to process and present materials to T-cells in the context of MHC Class II [19]. Also they have the ability to up-regulate co-stimulatory molecules including B7-1 and B7-2, which are needed to allow T-cell expansion following peptide presentation [19,20]. The up-regulation of these molecules may cause the release of IL-1, which increases inflammation at the local site of the liver [19]. Therefore, the secretion of cytokines and chemokines by SLECs in response to MAA-Alb stimulation, may result in the up-regulation of adhesion and co-stimulatory molecules, resulting in an inflammatory response in the livers of alcoholics.

Finally, SLECs have been shown to play a role in the activation of stellate cells. In studies by Jarnagin et al. [17], the EIIIA isoform of fibronectin has been shown to be released from activated SLECs, bind to stellate cells and induce the release of collagen. Thus, SLECs may play a very important role in the fibrotic process.
